# Learning Clinical Procedures Through Internet Digital Objects: Experience of Undergraduate Students Across Clinical Faculties

**DOI:** 10.2196/mededu.3866

**Published:** 2015-04-14

**Authors:** Tse Yan Li, Xiaoli Gao, Kin Wong, Christine Shuk Kwan Tse, Ying Yee Chan

**Affiliations:** ^1^ The University of Hong Kong Faculty of Dentistry Hong Kong China (Hong Kong)

**Keywords:** clinical skills, distance learning, dentistry, medicine, nursing

## Abstract

**Background:**

Various digital learning objects (DLOs) are available via the World Wide Web, showing the flow of clinical procedures. It is unclear to what extent these freely accessible Internet DLOs facilitate or hamper students’ acquisition of clinical competence.

**Objective:**

This study aimed to understand the experience of undergraduate students across clinical disciplines—medicine, dentistry, and nursing—in using openly accessible Internet DLOs, and to investigate the role of Internet DLOs in facilitating their clinical learning.

**Methods:**

Mid-year and final-year groups were selected from each undergraduate clinical degree program of the University of Hong Kong—Bachelor of Medicine and Bachelor of Surgery (MBBS), Bachelor of Dental Surgery (BDS), and Bachelor of Nursing (BNurs). All students were invited to complete a questionnaire on their personal and educational backgrounds, and their experiences and views on using Internet DLOs in learning clinical procedures. The questionnaire design was informed by the findings of six focus groups.

**Results:**

Among 439 respondents, 97.5% (428/439) learned a variety of clinical procedures through Internet DLOs. Most nursing students (107/122, 87.7%) learned preventive measures through Internet DLOs, with a lower percentage of medical students (99/215, 46.0%) and dental students (43/96, 45%) having learned them this way (both *P*<.001). Three-quarters (341/439, 77.7%) of students accessed DLOs through public search engines, whereas 93.2% (409/439) accessed them by watching YouTube videos. Students often shared DLOs with classmates (277/435, 63.7%), but rarely discussed them with teachers (54/436, 12.4%). The accuracy, usefulness, and importance of Internet DLOs were rated as 6.85 (SD 1.48), 7.27 (SD 1.53), and 7.13 (SD 1.72), respectively, out of a high score of 10.

**Conclusions:**

Self-exploration of DLOs in the unrestricted Internet environment is extremely common among current e-generation learners and was regarded by students across clinical faculties as an important supplement to their formal learning in the planned curriculum. This trend calls for a transformation of the educator’s role from dispensing knowledge to guidance and support.

## Introduction

The growing popularity of the Internet in the past two decades has entirely changed people’s lifestyles and the learning patterns of students around the world. As a new form of knowledge acquisition, Web-based learning has been advocated and incorporated widely as a supportive measure to the traditional ways of learning in classrooms [1,2]. It has also become an important part of health sciences education [3]. Although cumulative evidence does not support its higher effectiveness over traditional learning, Web-based learning has often been associated with several advantages, such as accessibility and convenience, cost-saving features, better acceptance, and higher student satisfaction, especially when combined with traditional teaching activities in a blended-learning setting [1,4,5]. Students’ modes of Web-learning are not limited to e-learning resources provided by their faculties or prescribed by the teaching staff, but also include spontaneous information seeking through the Internet—a learning pattern that is highly encouraged under the concept of self-directed learning [1,2,6].

For students who are pursuing careers as health care providers, acquiring competence in performing respective clinical procedures is a fundamental part of their professional training. The general public and students’ future employers would expect a high standard of clinical performance and patient management when they graduate. Traditionally, clinical procedures are explained, demonstrated, and practiced in the preclinical and clinical sessions through face-to-face, instructor-led learning. Student clinicians learn and refine their skills through observing and practicing on mannequins, virtual simulation, and clinical placements. Knowledge can also be obtained through many digital learning objects (DLOs), be it videos, animations, illustrations, or photos showing the flow of the procedures. It is believed that “multimedia instructional messages that are designed in light of how the human mind works are more likely to lead to meaningful learning than those that are not” [7]. Studies have shown better learning outcomes when audiovisual materials were used as compared with mere text materials [8]. Traditional teaching methods are comparatively passive in nature in bringing about understanding, retention, and application of information delivered, as active processing of materials is hindered [7]. A more dynamic approach is advocated, and together with the blooming usage of electronic appliances in the new generation, it is perhaps not too difficult to recognize the trend of dissemination of information in more innovative approaches utilizing information technologies.

Many DLOs for medical education purposes are available via the World Wide Web and are increasingly used by clinical leaners [2,9,10]. It is, however, largely unknown what students experience during this process and how these materials shape their clinical learning [1,11]. Understanding these factors will be useful for educators, students, and practitioners to improve their teaching systems or learning patterns. Previous studies are mainly directed at e-learning through materials provided by faculties [2-4]. Little is known about how students explore freely accessible materials on the Internet and use them for their clinical learning. Our previous qualitative study, using six focus groups consisting of undergraduate students, has captured a wide spectrum of students’ opinions toward Internet DLOs [12]. While there were many approving views supporting the unique roles of Internet DLOs, some concerns were raised regarding the use of these materials for their clinical learning [12].

Based on the findings of our qualitative study, this larger-scale quantitative study was carried out to understand the experience of undergraduate students across clinical disciplines—medicine, dentistry, and nursing—in using openly accessible Internet DLOs, and to investigate the impact of Internet DLOs on their clinical learning.

## Methods

### Digital Learning Objects

Internet DLOs were defined as digital learning materials (eg, videos, animations, graphic illustrations, and photos) that were openly accessible on public websites. Pure text materials were not included. The e-learning materials provided by students’ own faculties were not included in the scope of this study.

### Target Groups

This study targeted current undergraduate students in clinical faculties of the University of Hong Kong, which is the sole institution in Hong Kong dedicated to training dentists, and one of the two institutes providing degree programs in medicine and nursing. Following the education reform in 2012, undergraduate programs in Hong Kong’s universities have been extended by a year. Current students recruited before 2012 are still under the original 5-year Bachelor of Medicine and Bachelor of Surgery (MBBS) program, the 5-year Bachelor of Dental Surgery (BDS) program, and the 4-year Bachelor of Nursing (BNurs) program. There are two semesters in each academic year. Holding a first degree is not a requirement for admission to these clinical programs. The curricula in these three clinical programs are integrated, student centered, and inquiry based to promote students’ critical thinking skills and application of acquired knowledge. Early clinical contact is arranged in the first or second year. Clinical sessions gradually take up an increased proportion of their teaching hours until their final year of studies, when attachments to different departments in various hospitals are organized.

From each clinical degree program, mid-year students—MBBS III, BDS III, and BNurs II—and final-year students—MBBS V, BDS V, and BNurs IV—were selected for this study. All students enrolled in the selected years were eligible to join this study, regardless of their gender, age, secondary educational background (ie, local schools, international schools in Hong Kong, or overseas), and prior degree attainment (ie, first-degree holder or not). The protocol of this study was reviewed by the Institutional Review Board of the University of Hong Kong/Health Authority Hong Kong West Cluster. Ethical approval was obtained (reference number: UW13-020). The details of this study were explained to students through a participant information sheet. Written consent was obtained from each participating student.

### Questionnaire Design

A self-administered structured questionnaire was developed to collect the following participant information: (1) demographic profile (ie, age and gender), (2) secondary educational background, (3) prior degree attainment, (4) usage of, and access to, Internet DLOs, (5) procedures learned through Internet DLOs, (6) frequency and scenarios of using Internet DLOs, (7) sharing with peers and clarification with tutors/teachers, and (8) ratings on the accuracy, usefulness, and importance of Internet DLOs.

The formulation of questions was informed by the findings of our previous qualitative study (ie, focus groups with students) [12]. All questions were in English, which is the medium of instruction at this university, and were pretested among 4 students to ensure clarity. Out of a total of 13 questions, 12 were closed-ended, multiple-choice questions (MCQs) (see Multimedia Appendix 1). For one MCQ on learning preventive measures, the possible answers were tailor-made for each degree program so that the question was relevant to individual programs. Since clinical procedures were many, an open question was asked allowing students to fill in the procedures they learned through Internet DLOs.

### Participant Recruitment and Completion of Questionnaires

All students in the selected years of three degree programs were approached by several means: (1) MBBS III students during full class lectures, (2) MBBS V students during small group lectures, (3) BDS III students during Simulation Laboratory class, (4) BDS V students through their clinical group representatives, and (5) BNurs II students during full class lectures. Since BNurs IV students had clinical practice in groups of two and were scattered throughout different hospitals in Hong Kong, direct access to them was difficult. Hence, an invitation was posted in their class Facebook group through their class representative. A total of four reminders were posted. To further improve the response, the departmental office of the School of Nursing was approached and an invitation was sent to the university email accounts of all BNurs IV students through departmental circulars.

Participants in BNurs IV completed an electronic questionnaire posted online, whereas other participants completed a printed questionnaire at the venue where they were recruited. All questionnaires were completed anonymously. The completion of a questionnaire took approximately 5 minutes.

### Data Analysis

Data were analyzed using IBM Statistical Package for the Social Sciences (SPSS) version 20. Descriptive analysis was done on participants’ demographic profile, their educational background, and their experiences and views on using Internet DLOs to learn clinical procedures. Inferential analysis was conducted for identifying factors (ie, age, gender, secondary educational background, prior degree attainment, current degree program, and year of study) associated with students’ usage of, and opinions on, Internet DLOs. Parametric or nonparametric tests were used, as appropriate, for comparing means. The chi-square test was used for comparing proportions. Multivariate analysis was conducted for identifying factors affecting students’ ratings on Internet DLOs (linear regressions) and the use of Internet DLOs for learning preventive measures (logistic regressions) after controlling for other factors.

## Results

### Response Rate and Profiles of Participants

In total, 439 students participated in the questionnaire survey, including 218 (49.7%) MBBS students, 97 (22.1%) BDS students, and 124 (28.2%) BNurs students. The response rates for MBBS III, MBBS V, BDS III, BDS V, BNurs II, and BNurs IV students were 65.0% (104/160), 72.6% (114/157), 93% (52/56), 90% (45/50), 56.1% (101/180), and 12.8% (23/180), respectively.

The majority (371/439, 84.5%) of the participants were 20 to 23 years old. Males and females made up 41.7% (183/439) and 58.3% (256/439) of the participants, respectively. Out of the 439 participants, 351 (80.0%) completed their secondary education in local schools, while 4.8% (21/439) and 15.3% (67/439) of students graduated from international schools in Hong Kong and overseas schools, respectively. Out of 439 participants, 42 (9.6%) had obtained a first degree before joining the current clinical program.

### Usage of, and Access to, Internet Digital Learning Objects

Only 2.5% (11/439) of the participants had never used Internet DLOs, while the overwhelming majority (428/439, 97.5%) had experience in learning clinical procedures through Internet DLOs (see Table 1). The majority of the participants (409/439, 93.2%) accessed Internet DLOs from YouTube, and almost half of them (180/439, 41.0%) accessed Internet DLOs from other universities’ websites. Other sources included blogs (55/439, 12.5%), manufacturers’ guidelines (131/439, 29.8%), and other websites (118/439, 26.9%). Three-quarters of participants (341/439, 77.7%) found Internet DLOs through public search engines, while one-third (152/439, 34.6%) and one-quarter (126/439, 28.7%) received recommendations from classmates or teaching staff.

Students often used Internet DLOs before their first time performing a procedure (267/439, 60.8%), while one-quarter (113/439, 25.7%) accessed Internet DLOs after that time. Around half (241/439, 54.9%) used Internet DLOs to reinforce their clinical skills and 28.9% (127/439) used Internet DLOs to learn procedures that they rarely have the chance to practice. Over half (241/439, 54.9%) of the students used Internet DLOs to learn “some procedures.” One-third (159/439, 36.2%) used it for “few procedures,” while only 7.5% (33/439) of students used it for “most or all procedures.”

**Table 1:** Uses of, and access to, Internet digital learning objects.

Information about Internet DLOs^a^		Participants (n=439), n (%)^b^
**Usage**		
	Always	428 (97.5)
	Never	11 (2.5)
**Source(s)**		
	YouTube	409 (93.2)
	Blogs	55 (12.5)
	Manufacturers’ guidelines	131 (29.8)
	Other universities’ websites	180 (41.0)
	Other websites	118 (26.9)
**How students found Internet DLOs**		
	Recommendations from classmates	152 (34.6)
	Recommendations from teaching staff	126 (28.7)
	Public search engine	341 (77.7)
	Others	8 (1.8)
**Scenarios for use**		
	Before first time performing a procedure	267 (60.8)
	After first time performing a procedure	113 (25.7)
	To reinforce skills	241 (54.9)
	For some procedures I have rare chances to practice	127 (28.9)
**Frequency of use**		
	Few procedures	159 (36.2)
	Some procedures	241 (54.9)
	Most procedures	26 (5.9)
	All procedures	7 (1.6)

^a^Digital learning objects (DLOs).

^b^Percentages may add up to more than 100% since multiple choices were allowed.

### Procedures Learned

Clinical procedures learned by BDS students through Internet DLOs were mainly restoration (49/97, 51%), tooth preparation for crown or denture (46/97, 47%), oral surgery (36/97, 37%), preoperative preparation (31/97, 32%), and impression or facebow record (29/97, 30%) (see Table 2). Medical students mainly learned clinical examination (86/218, 39.4%), surgery (71/218, 32.6%), and catheter handling (62/218, 28.4%), whereas nursing students mainly learned catheter handling (63/124, 50.8%) and wound dressing (54/124, 43.5%) through Internet DLOs.

As for preventive procedures, about a quarter (26/96, 27%) of the dental students learned fluoride application, while some students learned fissure sealant placement (17/96, 18%), oral hygiene instructions (10/96, 10%), and prophylaxis (6/96, 6%). No dental student reported learning dietary counseling through Internet DLOs. Preventive measures learned by medical students included hygienic instructions (64/215, 29.8%), counseling on lifestyle (37/215, 17.2%), vaccination (28/215, 13.0%), and prenatal counseling (22/215, 10.2%). Nursing students often learned hygienic instructions (87/122, 71.3%), counseling on lifestyle (30/122, 24.6%), vaccination (21/122, 17.2%), and elderly care (25/122, 20.5%). Over half of the dental students (53/96, 55%) and medical students (116/215, 54.0%) never used Internet DLOs to learn preventive measures, whereas the percentage was 12.3% (15/122) among nursing students.

**Table 2:** Procedures and measures learned through Internet digital learning objects.

Type of procedure or measure			Participants, n (%)^a^
**Clinical procedures**			
	**MBBS** ^b^ **(n=218)**		
		Clinical examination	86 (39.4)
		Surgery	71 (32.6)
		Catheter handling	62 (28.4)
		Personal protective equipment	9 (4.1)
		Others (eg, endoscopy)	74 (33.9)
	**BDS** ^b^ **(n=97)**		
		Simple restorative work	49 (51)
		Crown/denture tooth preparation	46 (47)
		Oral surgery	36 (37)
		Preoperative preparation (eg, rubber dam)	31 (32)
		Impression/facebow record	29 (30)
		Others (eg, root debridement)	37 (38)
	**BNurs** ^b^ **(n=124)**		
		Catheter handling	63 (50.8)
		Wound dressing	54 (43.5)
		Personal protective equipment	24 (19.4)
		Clinical examination	16 (12.9)
		Surgery	13 (10.5)
		Others (eg, oral care)	45 (36.3)
**Preventive measures**			
	**MBBS (n=215)**		
		Hygienic instructions	64 (29.8)
		Counseling on lifestyle	37 (17.2)
		Vaccination	28 (13.0)
		Prenatal counseling	22 (10.2)
		Elderly care	7 (3.3)
		Others (eg, anti-drug abuse)	1 (0.5)
		None of the above	116 (54.0)
	**BDS (n=96)**		
		Fluoride application	26 (27)
		Fissure sealant	17 (18)
		Oral hygiene instruction	10 (10)
		Prophylaxis	6 (6)
		Dietary counseling	0 (0)
		None of the above	53 (55)
	**BNurs (n=122)**		
		Hygienic instructions	87 (71.3)
		Counseling on lifestyle	30 (24.6)
		Elderly care	25 (20.5)
		Vaccination	21 (17.2)
		Prenatal counseling	9 (7.4)
		Others (eg, psychiatric predischarge counseling)	2 (1.6)
		None of the above	15 (12.3)

^a^Percentages may add up to more than 100% since multiple choices were allowed.

^b^Bachelor of Medicine and Bachelor of Surgery (MBBS), Bachelor of Dental Surgery (BDS), Bachelor of Nursing (BNurs).

Multivariate analysis showed that, compared with nursing students, medical students (odds ratio [OR] 0.121, 95% CI 0.065-0.227) and dental students (OR 0.116, 95% CI 0.058-0.231) were less likely to learn preventive measures through Internet DLOs (*P*<.001) (see Table 3).

**Table 3:** Effect of program on learning of preventive measures.

Program	Learning preventive measures through Internet DLOs^a^, odds ratio (95% CI)^b^	*P*
BNurs^c^	1 (reference)	<.001
MBBS^c^	0.121 (0.065-0.227)	
BDS^c^	0.116 (0.058-0.231)	

^a^Digital learning objects (DLOs).

^b^Results were obtained through stepwise logistic regression. The dependent variables were “learning any preventive measure through Internet DLOs or not.” Independent variables entered were age, gender, degree program, year of study, secondary educational background, and prior degree attainment.

^c^Bachelor of Nursing (BNurs), Bachelor of Medicine and Bachelor of Surgery (MBBS), Bachelor of Dental Surgery (BDS).

### Sharing Internet Digital Learning Objects for Discussion and Clarification

Two-thirds (277/435, 63.7%) of students shared content of Internet DLOs with classmates, but only 12.4% (54/436) discussed DLOs with their teachers or clinical tutors (see Table 4). When the content of an Internet DLO contradicted with formal teaching, students mainly clarified with classmates (242/438, 55.3%) or tutors/teachers (245/438, 55.9%), or kept searching for other sources (198/438, 45.2%). A small proportion chose to trust (31/438, 7.1%) or ignore (65/438, 14.8%) the content in Internet DLOs without clarification.

**Table 4:** Sharing and clarification of Internet digital learning objects.

Sharing or clarification activities		Participants, n (%)^a^
**Share/discuss with classmates (n=435)**		
	Yes	277 (63.7)
	No	158 (36.3)
**Share/discuss with teachers (n=436)**		
	Yes	54 (12.4)
	No	382 (87.6)
**Action when Internet DLOs** ^b^ **contradict formal teaching (n=438)**		
	Trust Internet DLOs	31 (7.1)
	Ignore Internet DLOs	65 (14.8)
	Discuss with classmates	242 (55.3)
	Clarify with tutors/teachers	245 (55.9)
	Keep searching for other sources	198 (45.2)

^a^Percentages may add up to more than 100% since multiple choices were allowed.

^b^Digital learning objects (DLOs).

### Rating of Internet Digital Learning Objects

Students’ mean ratings on the *accuracy*, *usefulness* and *importance* of Internet DLOs were 6.85 (SD 1.48), 7.27 (SD 1.53), and 7.13 (SD 1.72), respectively, out of a high score of 10 (see Table 5). MBBS and BDS students gave higher ratings on the *accuracy* of Internet DLOs as compared with nursing students (*P*=.034 and .044, respectively). No significant difference was found among three degree programs in students’ mean ratings on the *usefulness* and *importance* of Internet DLOs (*P*=.213 and .908, respectively).

**Table 5:** Rating of Internet digital learning objects.

DLO^a^ characteristic	Rating, mean (SD)			
	MBBS^b^ (n=218)	BDS^b^ (n=97)	BNurs^b^ (n=124)	Total (n=439)
Accuracy^c^	6.96 (1.40)	7.02 (1.23)	6.54 (1.74)	6.85 (1.48)
Usefulness	7.39 (1.53)	7.27 (1.30)	7.05 (1.66)	7.27 (1.53)
Importance	7.17 (1.82)	7.11 (1.55)	7.07 (1.69)	7.13 (1.72)

^a^Digital learning object (DLO).

^b^Bachelor of Medicine and Bachelor of Surgery (MBBS), Bachelor of Dental Surgery (BDS), Bachelor of Nursing (BNurs).

^c^Rating on *accuracy* of Internet DLOs was significantly higher among MBBS and BDS students than among BNurs students (*P*=.034 and .044, respectively).

Multivariate analysis showed that, compared with their counterparts, nursing students and female students rated the *accuracy* and *usefulness* of Internet DLOs more unfavorably (*P*=.010 and .018, respectively) (see Table 6).

**Table 6:** Factors affecting rating of Internet digital learning objects.

Factor affecting DLO^a^ rating		B^b^ (95% CI)	*P*
**Rating on** *accuracy* **of Internet DLOs**			
	Constant	6.986 (6.820-7.153)	<.001
	Nursing students	-0.419 (-0.737 to -0.100)	.010
**Rating on** *usefulness* **of Internet DLOs**			
	Constant	7.863 (7.362-8.364)	<.001
	Female students	-0.366 (-0.668 to -0.064)	.018
Rating on *importance* of Internet DLOs		N/A^c^	

^a^Digital learning object (DLO).

^b^Regression coefficient (B): results were obtained through stepwise multiple linear regression. The dependent variables were students’ ratings on Internet DLO characteristics. Independent variables entered were age, gender, degree program, year of study, secondary educational background, and prior degree attainment.

^c^Not applicable (N/A): no associated factor identified.

## Discussion

### Principal Findings

The World Wide Web has opened up new horizons for learners at all levels. As in many other fields of education, clinical students’ self-exploration of learning resources in the unrestricted Internet environment is very common. This is supported by our finding that almost all (428/439, 97.5%) students used Internet DLOs to facilitate their clinical learning. In addition to the ‘‘see one, do one, teach one’’ apprenticeship model for medical education [13,14], students often “Google many” to consolidate their clinical skills. This may prepare students to perform procedures safely and reduce the chance of preventable harm to patients [15].

Students reported learning a wide range of clinical procedures through Internet DLOs. Learning preventive measures through Internet DLOs was more common among nursing students than with medical and dental students. This may be due to the fact that medical doctors and dentists are increasingly delegating preventive work to auxiliary staff, whereas nurses tend to take up the role of educating patients. Dental students learned fluoride application, dental sealants placement, and oral hygiene instructions from Internet DLOs. Nevertheless, no dental student reported learning dietary counseling through Internet DLOs, although it is regarded as a main component for patient counseling in order to prevent oral diseases [16,17]. This might reflect the lower priority that students give to dietary counseling, or their underestimation of the skills required for effective dietary counseling. To the best of our knowledge, comparisons of Internet learning experiences of clinical students across various disciplines have not been reported previously. The differences among medical, dental, and nursing students can be further investigated in other populations.

Our findings suggested that Internet DLOs have become an important channel for students to connect to the international learning community. The Internet breaks the isolation of learners and enables learning interactions that were not possible before, such as the coupling of novices with experts from around the world, the opportunity to communicate with a world audience, and the ability to coconstruct knowledge and negotiate meaning [4,7]. Learning clinical procedures through Internet DLOs is not only relevant at the undergraduate education stage. It can be anticipated that this mode of learning will stay with students throughout their professional lives as an alternative method to gain procedural experience and to update their clinical skills.

Despite several advantages of technology-enhanced learning, such as its economic benefit, high efficiency, and easy and timely access, there has been a long-standing debate regarding whether media can influence learning [18,19]. Some believe that both the medium and instructional methods influence the ways that learners process information and construct knowledge [18,20]. However, many accept the assertion that media are mere vehicles that deliver instruction, and what influences learning is the instructional method underlying the medium employed [19,21]. From either side of the argument, the content and the instructional method are considered important for learning to occur. Given the wide use of Internet DLOs and their often uncensored nature, the question is raised regarding how to actively engage faculties in developing and selecting high-quality DLOs and in providing needed guidance to students [9]. Providing teachers with a framework, appropriate tools, and concrete assistance may help them use DLOs to exchange ideas at the international level.

Although the younger generation possesses a certain level of computer literacy, our survey showed that students’ searches for learning resources were predominately through public search engines, implying their limited ability to locate information [22,23]. Support from teaching, library, and information technology (IT) staff may be needed in order for students to take advantage of the possibilities that the Internet offers and be able to retrieve, evaluate, and synthesize information critically and effectively [24]. Although there is a myriad of videos and other DLOs on the Web, the majority are not developed by an accredited body or endorsed by an institution. Clearly stated learning objectives and outcomes are often lacking. Our findings showed that students’ ratings on the accuracy of Internet DLOs was only 6.85 out of 10. This highlighted students’ awareness and concerns of the low quality of a considerable amount of online material, which was observed in a previous study examining the quality of YouTube videos on the topic of medical science [9].

Educators, therefore, carry the role of guiding students in selecting quality and up-to-date materials to assist their learning. Collective efforts have been made to develop peer-reviewed learning resource banks [25-27]. Linking them to popular public search sites, for example, by creating a YouTube channel or iTunes app, might help increase the searchability of these websites and steer students to these reliable sources [28].

### Limitations

Various means were attempted in this study to approach all students in the three degree programs. This contributed to a high response from dental students and a reasonable response from medical students and nursing mid-year students. However, the response from nursing final-year students to the online questionnaire was low, despite various efforts. This might have introduced some bias into some of our findings concerning final-year nursing students. Although this study involved students in three clinical programs, data were collected from only one university that adopts a student-centered and inquiry-based learning system. In universities using a traditional didactic teaching method, students may hold different views, which are yet to be explored in future studies. In addition, learning through Internet DLOs may not be equally relevant for students in developing and underdeveloped countries, where easy and free access to Internet resources is not possible.

### Conclusions

Our study showed that Internet DLOs are a commonly used channel for learning clinical procedures among undergraduate students in dentistry, medicine, and nursing. They are regarded by undergraduate students across clinical faculties as useful and important supplements to their formal learning in the planned curriculum. Self-exploration of learning resources in the unrestricted Internet environment has a profound impact on the clinical training of e-generation learners. This trend calls for a transformation of the educator’s role from dispensing knowledge to guidance and support.

## Appendix

Multimedia Appendix 1 Study questionnaire.

## Abbreviations

BDS: Bachelor of Dental Surgery
BNurs: Bachelor of Nursing
DLO: digital learning object
IT: information technology
MBBS: Bachelor of Medicine and Bachelor of Surgery
MCQ: multiple-choice question
OR: odds ratio
SPSS: Statistical Package for the Social Sciences
