# Supplementary material for: Learning Clinical Procedures Through Internet Digital Objects: Experience of Undergraduate Students Across Clinical Faculties
Source: JMIR Med Educ. 2015 Apr 14;1(1):e1. doi: 10.2196/mededu.3866 (PMC5041352; doi:10.2196/mededu.3866)
Supplement: Multimedia Appendix 1 [file mededu_v1i1e1_app1.pdf]

III. Questionnaire for BDS students

Code: BDS_____

Questionnaire

*This questionnaire is for understanding students' experience and opinions on using Internet Visual Resources (**other than those provided by own faculty**) for learning clinical procedures. Your participation is very important to us. Thank you for your cooperation.*

Age: _______ Years   Gender: □ M   □ F

Current Year of Study: □ BDS III   □ BDS V

Secondary education background: (you may choose more than one answer)
□ Local schools   □ International schools in Hong Kong
□ Overseas: ____________ (please specify country)

Are you a degree holder?
□ Yes, Major in ______________   □ No

You may choose MORE THAN ONE option for the questions below.
Please tick appropriate box(es):

1. Have you ever learnt any **clinical procedure** from any type of Internet visual resources?
   - □ Videos
   - □ Animation
   - □ Graphical illustration (Pictures showing procedure stepwise)
   - □ Photos showing sequence of procedure
   - □ Others: ____________________ (Please specify)
   - □ I have never used Internet visual resources for learning clinical procedures

2. From what sources did you get access to the Internet visual materials?
   - □ YouTube
   - □ Blogs
   - □ Manufacturer's guidelines
   - □ Other universities' websites
   - □ Other websites

3. Which **clinical procedures** did you ever learn through Internet visual resources?

   ________________, ________________, ________________

4. Did your learning through Internet visual resources include any of the following **preventive measures**?
   - □ Fissure sealant
   - □ Prophylaxis
   - □ Fluoride application
   - □ OHI
   - □ Dietary counseling
   - □ Others: ____________________ (Please specify)
   - □ None of the above

(Please turn over)

5. Under what scenarios would you use these resources?
   - □ Before 1st time performing a procedure
   - □ After 1st time performing a procedure
   - □ To reinforce skills
   - □ For specialty procedures I have rare chances to practise (e.g. re-endo, implant)
   - □ Others: ____________________ (Please specify)

6. You would use these resources for:
   - □ Few procedures
   - □ Some procedures
   - □ Most procedures
   - □ All procedures

7. How did you get to know about such Internet visual resources?
   - □ Recommendations from classmates
   - □ Recommendations from teaching staff
   - □ Search engine
   - □ Others: ____________________ (Please specify)

8. Have you ever shared / discussed the online resources with any **classmate**?

   □ Yes □ No

9. Have you ever shared / discussed the online resources with any **teacher**?

   □ Yes □ No

10. How much do you think the procedures demonstrated are **accurate** (i.e. in line with well accepted practices)? (Please circle)

    (**not at all**) 0 ---1 --- 2 ---3 --- 4 --- 5 --- 6 --- 7 --- 8 --- 9 --- 10 (**very much**)

11. How will you rate the **usefulness** of the Internet visual resources in general? (Please circle)

    (**least** useful) 0 ---1 --- 2 ---3 --- 4 --- 5 --- 6 --- 7 --- 8 --- 9 --- 10 (**most** useful)

12. How will you rate the **importance** of the Internet visual resources as a <u>supplement</u> to learning? (Please circle)

    (**least** important) 0----1----2----3----4----5----6----7----8----9----10 (**most** important)

13. If you find these resources contradict to what you learn from textbooks, faculty resources and clinical instructors, what will you do?
    - □ Trust Internet visual resources
    - □ Ignore the Internet visual resources
    - □ Discuss with classmates
    - □ Clarify with tutors / teachers
    - □ Keep searching for other sources
    - □ Others: ____________________ (Please specify)

------------------------------------ ***End of questionnaire*** ------------------------------------

IV. Questionnaire for MBBS students

Code: MBBS_____

Questionnaire

*This questionnaire is for understanding students' experience and opinions on using Internet Visual Resources (**other than those provided by own faculty**) for learning clinical procedures. Your participation is very important to us. Thank you for your cooperation.*

Age: _______ Years Gender: □ M □ F

Current Year of Study: □ MBBS III □ MBBS V

Secondary education background: (you may choose more than one answer)
□ Local schools □ International schools in Hong Kong
□ Overseas: ____________ (please specify country)

Are you a degree holder?
□ Yes, Major in ______________ □ No

You may choose MORE THAN ONE option for the questions below.
Please tick the appropriate box(es):

1. Have you ever learnt any **clinical procedure** from any type of Internet visual resources?
    - □ Videos
    - □ Animation
    - □ Graphical illustration (Pictures showing procedure stepwise)
    - □ Photos showing sequence of procedure
    - □ Others: ____________________ (Please specify)
    - □ I have never used Internet visual resources for learning clinical procedures

2. From what sources did you get access to the Internet visual materials?
    - □ YouTube
    - □ Blogs
    - □ Manufacturer's guidelines
    - □ Other universities' websites
    - □ Other websites

3. Which **clinical procedures** did you ever learn through Internet visual resources?

    _________________, _________________, _________________

4. Did your learning through Internet visual resources include any of the following **preventive measures**?
    - □ Hygienic instructions
    - □ Counseling on lifestyle (e.g. smoking, diet, exercise)
    - □ Vaccination
    - □ Prenatal counseling
    - □ Elderly care
    - □ Others: ____________________ (Please specify)
    - □ None of the above

(Please turn over)

5. Under what scenarios would you use these resources?
   - □ Before 1st time performing a procedure
   - □ After 1st time performing a procedure
   - □ To reinforce skills
   - □ For specialty procedures I have rare chances to practice (e.g. surgery, biopsy sampling)
   - □ Others: ____________________ (Please specify)

6. You would use these resources for:
   - □ Few procedures
   - □ Some procedures
   - □ Most procedures
   - □ All procedures

7. How did you get to know about such Internet visual resources?
   - □ Recommendations from classmates
   - □ Recommendations from teaching staff
   - □ Search engine
   - □ Others: ____________________ (Please specify)

8. Have you ever shared / discussed the online resources with any **classmate**?
   □ Yes □ No

9. Have you ever shared / discussed the online resources with any **teacher**?
   □ Yes □ No

10. How much do you think the procedures demonstrated are **accurate** (i.e. in line with well accepted practices)? (Please circle)

    (**not at all**) 0 ---1 --- 2 ---3 --- 4 --- 5 --- 6 --- 7 --- 8 --- 9 --- 10 (**very much**)

11. How will you rate the **usefulness** of the Internet visual resources in general? (Please circle)

    (**least** useful) 0 ---1 --- 2 ---3 --- 4 --- 5 --- 6 --- 7 --- 8 --- 9 --- 10 (**most** useful)

12. How will you rate the **importance** of the Internet visual resources as a <u>supplement</u> to learning? (Please circle)

    (**least** important) 0----1----2----3----4----5---6----7----8----9----10 (**most** important)

13. If you find these resources contradict to what you learn from textbooks, faculty resources and clinical instructors, what will you do?
    - □ Trust Internet visual resources
    - □ Ignore the Internet visual resources
    - □ Discuss with classmates
    - □ Clarify with tutors / teachers
    - □ Keep searching for other sources
    - □ Others: ____________________ (Please specify)

----------------------------------- ***End of questionnaire*** -----------------------------------

V. Questionnaire for BNurs students

Code: BNurs_____

Questionnaire

*This questionnaire is for understanding students' experience and opinions on using Internet Visual Resources (**other than those provided by own faculty**) for learning clinical procedures. Your participation is very important to us. Thank you for your cooperation.*

Age: _______ Years   Gender: □ M   □ F

Current Year of Study: □ BNurs II   □ BNurs IV

Secondary education background: (you may choose more than one answer)
□ Local schools   □ International schools in Hong Kong
□ Overseas: ____________ (please specify country)

Are you a degree holder?
□ Yes, Major in ______________   □ No

You may choose MORE THAN ONE option for the questions below.
Please tick the appropriate box(es):

1. Have you ever learnt any **clinical procedure** from any type of Internet visual resources?
   - □ Videos
   - □ Animation
   - □ Graphical illustration (Pictures showing procedure stepwise)
   - □ Photos showing sequence of procedure
   - □ Others: ____________________ (Please specify)
   - □ I have never used Internet visual resources for learning clinical procedures

2. From what sources did you get access to the Internet visual materials?
   - □ YouTube
   - □ Blogs
   - □ Manufacturer's guidelines
   - □ Other universities' websites
   - □ Other websites

3. Which **clinical procedures** did you ever learn through Internet visual resources?

   ________________, ________________, ________________

4. Did your learning through Internet visual resources include any of the following **preventive measures**?
   - □ Hygienic instructions
   - □ Counseling on lifestyle (e.g. smoking, diet, exercise)
   - □ Vaccination
   - □ Prenatal counseling
   - □ Elderly care
   - □ Others: ____________________ (Please specify)
   - □ None of the above

(Please turn over)

5. Under what scenarios would you use these resources?
   - □ Before 1st time performing a procedure
   - □ After 1st time performing a procedure
   - □ To reinforce skills
   - □ For some procedures I have rare chances to practice
   - □ Others: ____________________ (Please specify)

6. You would use these resources for:
   - □ Few procedures
   - □ Some procedures
   - □ Most procedures
   - □ All procedures

7. How did you get to know about such Internet visual resources?
   - □ Recommendations from classmates
   - □ Recommendations from teaching staff
   - □ Search engine
   - □ Others: ____________________ (Please specify)

8. Have you ever shared / discussed the online resources with any **classmate**?
   □ Yes □ No

9. Have you ever shared / discussed the online resources with any **teacher**?
   □ Yes □ No

10. How much do you think the procedures demonstrated are **accurate** (i.e. in line with well accepted practices)? (Please circle)

    (**not at all**) 0 ---1 --- 2 ---3 --- 4 --- 5 --- 6 --- 7 --- 8 --- 9 --- 10 (**very much**)

11. How will you rate the **usefulness** of the Internet visual resources in general? (Please circle)

    (**least** useful) 0 ---1 --- 2 ---3 --- 4 --- 5 --- 6 --- 7 --- 8 --- 9 --- 10 (**most** useful)

12. How will you rate the **importance** of the Internet visual resources as a <u>supplement</u> to learning? (Please circle)

    (**least** important) 0----1----2----3----4----5----6----7----8----9----10 (**most** important)

13. If you find these resources contradict to what you learn from textbooks, faculty resources and clinical instructors, what will you do?
    - □ Trust Internet visual resources
    - □ Ignore the Internet visual resources
    - □ Discuss with classmates
    - □ Clarify with tutors / teachers
    - □ Keep searching for other sources
    - □ Others: ____________________ (Please specify)

------------------------------------ ***End of questionnaire*** ------------------------------------
